# Gender gap in deep brain stimulation for Parkinson’s disease

**DOI:** 10.1038/s41531-022-00305-y

**Published:** 2022-04-20

**Authors:** Stefanie T. Jost, Lena Strobel, Alexandra Rizos, Philipp A. Loehrer, Keyoumars Ashkan, Julian Evans, Franz Rosenkranz, Michael T. Barbe, Gereon R. Fink, Jeremy Franklin, Anna Sauerbier, Christopher Nimsky, Afsar Sattari, K. Ray Chaudhuri, Angelo Antonini, Lars Timmermann, Pablo Martinez-Martin, Monty Silverdale, Elke Kalbe, Veerle Visser-Vandewalle, Haidar S. Dafsari, EUROPAR and the International Parkinson and Movement Disorders Society Non-Motor Parkinson’s Disease Study Group

**Affiliations:** 1 Department of Neurology, University of Cologne, Faculty of Medicine and University Hospital Cologne, Cologne, Germany; 2 Parkinson Foundation International Centre of Excellence, King’s College Hospital, London, UK; 3 Department of Neurology, University Hospital Giessen and Marburg, Campus Marburg, Marburg, Germany; 4 Department of Neurology and Neurosurgery, Salford Royal NHS Foundation Trust, Manchester Academic Health Science Centre, University of Manchester, Greater Manchester, UK; 5 Cognitive Neuroscience, Institute of Neuroscience and Medicine (INM-3), Research Centre Jülich, Jülich, Germany; 6 Institute of Medical Statistics and Computational Biology (IMSB), University of Cologne, Cologne, Germany; 7 Department of Neurosurgery, University Marburg, Marburg, Germany; 8 University of Cologne, Faculty of Humanities, Cologne, Germany; 9 German Association of Women Engineers, Cologne, Germany; 10 Institute of Psychiatry, Psychology and Neuroscience, King’s College London, London, UK; 11 NIHR Mental Health Biomedical Research Centre and Dementia Biomedical Research Unit, South London and Maudsley NHS Foundation Trust and King’s College London, London, UK; 12 Parkinson and Movement Disorders Unit, Department of Neurosciences (DNS), University of Padua, Padova, Italy; 13 Center for Networked Biomedical Research in Neurodegenerative Diseases (CIBERNED), Carlos III Institute of Health, Madrid, Spain; 14 University of Cologne, Faculty of Medicine, Medical Psychology, Neuropsychology and Gender Studies & Center for Neuropsychological Diagnostics and Interventions (CeNDI), Cologne, Germany; 15 Department of Stereotactic and Functional Neurosurgery, Faculty of Medicine and University Hospital Cologne, University of Cologne, Cologne, Germany

**Keywords:** Parkinson's disease, Quality of life

## Abstract

Previous studies have shown less access to deep brain stimulation (DBS) for Parkinson’s disease (PD) in women compared to men raising concerns about a potential gender gap resulting from nonclinical factors or gender differences in clinical efficacy for postoperative quality of life (QoL), motor, and nonmotor symptoms (NMS) outcomes. This was a cross-sectional and a longitudinal, prospective, observational, controlled, quasi-experimental, international multicenter study. A total sample size of 505 consisted of 316 consecutively referred patients for DBS indication evaluation at the University Hospital Cologne (01/2015–09/2020) and 189 consecutively treated patients at DBS centers in the University Hospitals Cologne and Marburg, Salford’s Royal Hospital Manchester, and King’s College Hospital London. In the cross-sectional cohort, we examined gender proportions at referral, indication evaluations, and DBS surgery. In the longitudinal cohort, clinical assessments at preoperative baseline and 6-month follow-up after surgery included the PD Questionnaire-8, NMSScale, Scales for Outcomes in PD-motor scale, and levodopa-equivalent daily dose. Propensity score matching resulted in a pseudo-randomized sub-cohort balancing baseline demographic and clinical characteristics between women with PD and male controls. 316 patients were referred for DBS. 219 indication evaluations were positive (women *n* = 102, respectively *n* = 82). Women with PD were disproportionally underrepresented in referrals compared to the general PD population (relative risk [RR], 0.72; 95%CI, 0.56–0.91; *P* = 0.002), but more likely to be approved for DBS than men (RR, 1.17; 95%CI, 1.03–1.34; *P* = 0.029). Nonetheless, their total relative risk of undergoing DBS treatment was 0.74 (95%CI, 0.48–1.12) compared to men with PD. At baseline, women had longer disease duration and worse dyskinesia. Exploring QoL domains, women reported worse mobility and bodily discomfort. At follow-up, all main outcomes improved equally in both genders. Our study provides evidence of a gender gap in DBS for PD. Women and men with PD have distinct preoperative nonmotor and motor profiles. We advocate that more focus should be directed toward the implementation of gender equity as both genders benefit from DBS with equal clinical efficacy. This study provides Class II evidence of beneficial effects of DBS in women with PD compared to male controls.

## Introduction

Deep brain stimulation (DBS) is an effective treatment in advanced Parkinson’s disease (PD) improving quality of life (QoL)^1,2^, motor^3^, and nonmotor symptoms (NMS)^4–6^. PD affects men more frequently than women with an overall prevalence gender-ratio of 1.48:1 (M:F)^7–9^. In advanced stages of PD, women are at higher risk than men to develop motor complications, such as dyskinesia or motor fluctuations and manifest different nonmotor profiles than men^10,11^. Previous studies have shown disparities in access to DBS between men and women as women are less likely to undergo DBS^10,12,13^, which is out of proportion to prevalence data^8^. However, it is unclear, at which key steps from referral through indication evaluation until surgical procedures the disadvantages arise for women with PD. Furthermore, little is known about gender-related differences in postsurgical outcomes or distinct nonmotor and motor profiles that could explain this ‘gender gap’. In particular, gender differences in nonmotor outcomes following DBS have not been systematically investigated yet. Therefore, this study examined (1) gender proportions at key steps from referral for indication evaluations until DBS surgery and (2) gender differences at preoperative baseline and in postoperative outcomes at 6-month follow-up with respect to QoL, nonmotor, and motor symptoms. We hypothesized that (1) the gender gap at these key steps cumulates to an overall disadvantage for women with PD and (2) that there are distinct nonmotor and motor profiles in men and women undergoing DBS and that nonetheless both genders postoperatively experience beneficial QoL, motor, and nonmotor effects.

## Results

### Gender ratio in Parkinson’s disease: from indication evaluation to deep brain stimulation surgery

**Fig. 1:**
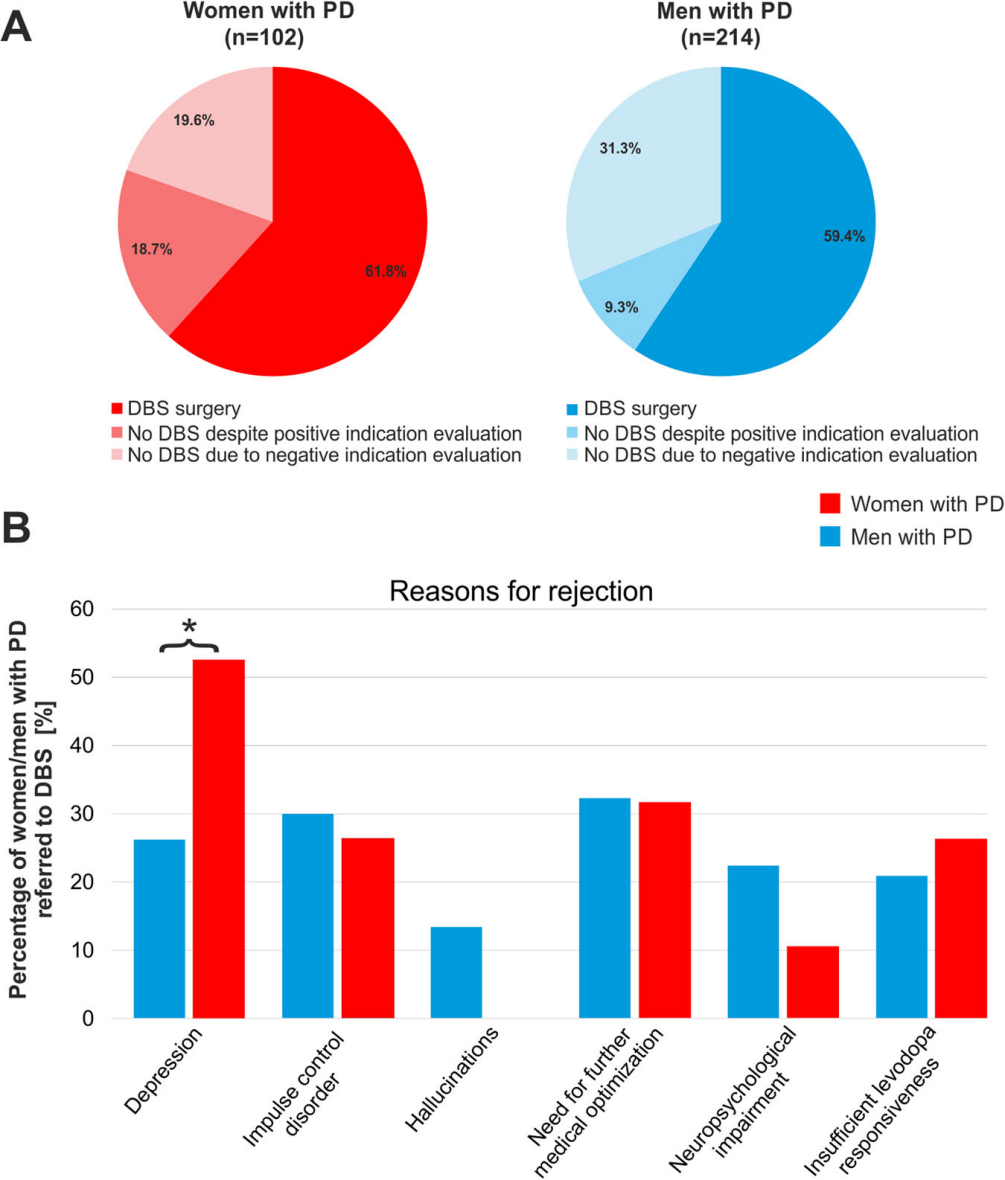
Gender ratio and reasons for not receiving deep brain stimulation in the cross-sectional cohort. In (**A**), pie charts illustrate ratios of women (left) and men (right) with Parkinson’s disease who underwent DBS surgery, or did not undergo DBS surgery either despite positive indication evaluation or due to negative indication evaluation. In (**B**), bar charts illustrate the percentage of women and men with PD rejected for different reasons in DBS indication evaluations. 53 patients had one reason for rejection, 29 patients had at least two reasons for rejection, 5 patients had other reasons for rejection (mania, pre-existing orthopedic or cardiovascular conditions). The black star represents significantly more rejections due to depression in women than in men with PD. DBS Deep brain stimulation, PD Parkinson’s disease.

In the cross-sectional cohort, 316 patients were referred for DBS indication evaluations at the University Hospital Cologne during the time period of January 2015 until September 2020 (see Table 1 and Fig. 1, women: *n* = 102). The gender ratio men:women was 2.1:1 (32% women). The proportion of women with PD referred for DBS indication evaluations was significantly lower than in the known general PD population of 1.48:1^7^ (40% women; one-sample binomial test, *P* = 0.002), resulting in a 0.72 relative risk (RR, 0.72; 95%CI, 0.56–0.91) of referral for women compared to men with PD.

**Table 1:** Gender ratios at indication evaluations, approval for deep brain stimulation and surgical procedures in the cross-sectional cohort.

Steps to DBS surgery	Women	Men	Total	*P*	Relative risk [95% CI]
Referred for DBS indication evaluation	102	214	316	**0.002**^c^	0.72 [0.56; 0.91]
Positive indication evaluation	82 (80.4%)^a^	147 (68.7%)^a^	229 (72.5%)^a^	**0.029**^d^	1.17 [1.03; 1.34]
DBS surgery	63 (76.8%)^b^	127 (86.4%)^b^	190 (83.0%)^b^	0.065^e^	0.89 [0.78; 1.02]

Significant results are highlighted in bold font. The total relative risk of DBS treatment for women with Parkinson’s disease compared to men was 0.73 (95% CI, 0.48; 1.12).

*CI* confidence interval, *DBS* deep brain stimulation.

^a^Percentage of patients referred for DBS indication evaluation.

^b^Percentage of patients with positive indication evaluation.

^c^Binomial test comparison of gender ratios of prevalence data and patients referred for DBS indication evaluation.

^d^Chi² test for gender ratio in patients with positive indication evaluation compared to patients referred for DBS indication evaluation.

^e^Chi² test for gender ratio in DBS surgery compared to positive indication evaluation.

229 patients were approved for DBS in the multidisciplinary indication evaluations with a gender ratio of 1.8:1 (women *n* = 82). Of these, 190 patients underwent DBS surgery with a gender ratio of 2.0:1 (women *n* = 63). Female gender was associated with an approval decision for DBS, *P* = 0.029, the probability of approval was 17% higher in women with PD (RR, 1.17; 95%CI, 1.03–1.34).

Indication evaluations were negative in 20 women with PD and 67 men with PD (22% women). The main reasons for negative indication assessments were: clinically relevant neuropsychological impairment (*n* = 17, women: *n* = 2) and neuropsychiatric symptoms, such as depression (*n* = 27, women: *n* = 10), impulse control disorders not depending on dopaminergic medication (*n* = 25, women: *n* = 5), and hallucinations (*n* = 9, women: *n* = 0). Other reasons were an insufficient levodopa responsiveness (*n* = 19, women: *n* = 5) or a need for further medical optimization (*n* = 27, women: *n* = 6). Less frequent reasons for negative indications assessments were abuse disorders, mania, diagnosis of atypical PD, and a high risk for intraoperative bleedings because of previous illnesses. The proportion of rejections due to clinically relevant depression was higher for women with PD (*P* = 0.037). No gender differences were observed for other rejection reasons (all *P* > 0.05).

Among patients approved for DBS, the proportion of women who eventually underwent DBS surgery was smaller on trend level (RR, 0.89, m; 95%CI, 1.03–1.34; *P* = 0.065). The probability of undergoing DBS after positive indication evaluations was 11% lower in women with PD. The total relative risk of undergoing DBS treatment for women compared to men with PD was 0.73 (95%CI, 0.48–1.12, see Table 1). The reasons for not undergoing DBS surgery despite positive indication evaluations were: patient wish for an additional period of reflection (*n* = 10, women: *n* = 3), patient preference of further medical optimization (*n* = 8, women: *n* = 3), newly developed or worsened preexisting comorbid diseases (*n* = 9, women: *n* = 8), language barrier (*n* = 1, women: *n* = 1), and personal reasons undisclosed by patients (*n* = 11, women: *n* = 4). Chi-squared statistics showed no significant association between gender and these reasons.

DBS targeted the subthalamic nucleus in 157 patients (55 women), the internal segment of the globus pallidus in 18 patients (3 women), and the ventral intermediate nucleus of the thalamus in 15 patients (5 women). We observed no significant relationship between gender and DBS target (*P* > 0.05).

### Clinical differences of men and women with Parkinson’s disease undergoing deep brain stimulation

Clinical assessments were conducted in a longitudinal multicenter cohort of 189 patients (women: *n* = 68, 36%). The mean age at preoperative baseline was 62.3 years ±8.5 and the mean time to follow-up was 0.5 ± 0.2 years.

### Baseline characteristics of the original cohort

**Fig. 2:**
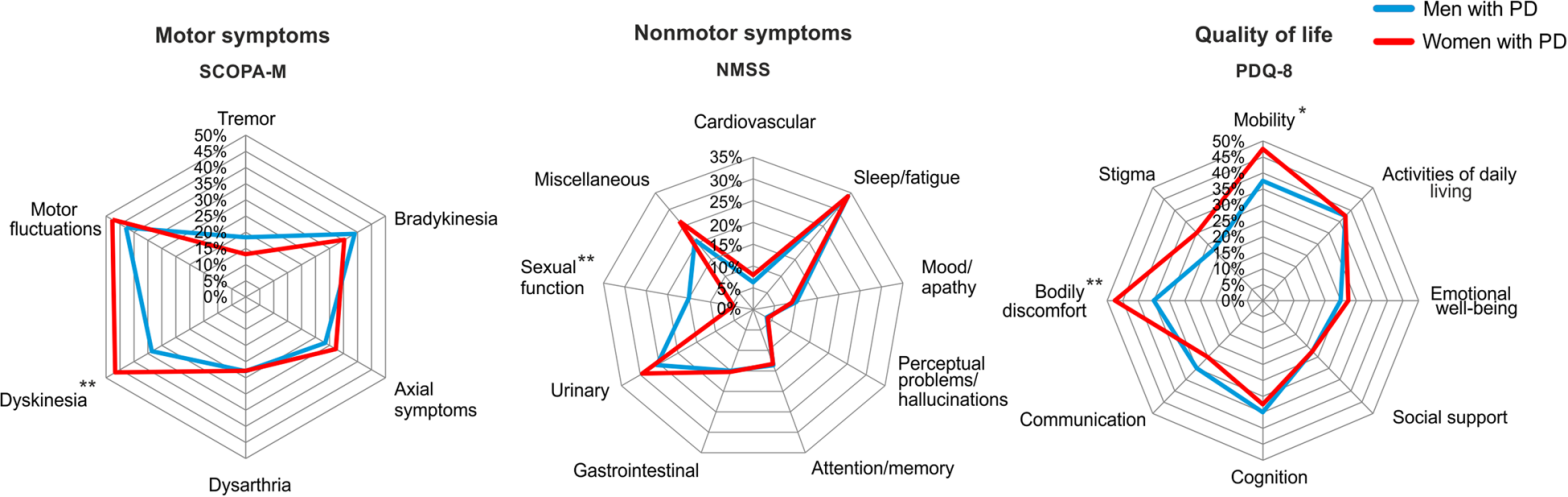
Preoperative gender differences in the original longitudinal cohort. Figure 2 illustrates motor (left), nonmotor (middle), and quality of life (right) domains in women and men with Parkinson’s disease undergoing bilateral deep brain stimulation. NMSS Non-motor Symptom Scale, PD Parkinson’s disease, PDQ-8 Parkinson’s Disease Questionnaire-8, SCOPA-M Scales for Outcomes in Parkinson’s Disease-motor scale. Significant differences between women and men at preoperative baseline highlighted with: * for *P* < 0.05. ** for *P* < 0.01.

Women with PD had a longer disease duration (∆2.5 years; 95%CI, 1.0–4.0; *P* = 0.001; see Table 2) and more severe dyskinesia (∆13.1; 95%CI, 4.2–22.0; *P* = 0.001). No significant gender differences were observed for the PDQ-8 SI, NMSS total score, and LEDD. Exploring PDQ-8 and NMSS domains (see Fig. 2), we observed that women with PD experienced worse PDQ ‘mobility’ (∆0.3; 95%CI, 0.0–0.7; *P* = 0.044) and ‘bodily discomfort’ (∆0.6; 95%CI 0.2–0.9; *P* = 0.002), whereas men with PD experienced worse NMSS ‘sexual functions’ (women: median 0.0, IQR [interquartile range], 0.0–0.0; men: median 0.0, IQR 0.0–6.0; *P* = 0.001).

**Table 2:** Baseline characteristics of women and men with Parkinson’s disease in the original longitudinal cohort.

	Women			Men			Women vs. Men	
	*n*	Mean	SD	*n*	Mean	SD	*P*	∆ [95% CI]
*Age*	68	62.7	8.5	121	62.0	8.4	0.624	−0.6 [−3.2; 1.9]
*Disease duration*	68	11.9	5.2	120	9.4	4.2	**0.001**	−2.5 [−4.0; −1.0]
PDQ-8 SI	68	34.3	15.6	115	30.5	16.5	0.129	−3.8 [−8.6; 1.1]
Mobility	68	1.9	1.0	115	1.5	1.2	**0.044**	−0.3 [−0.7; 0.0]
Activities of daily living	68	1.5	1.3	115	1.5	1.2	0.970	0.0 [−0.4; 0.4]
Emotional well-being	68	1.1	0.9	115	1.0	0.9	0.240	−0.2 [−0.4; 0.1]
Social support	68	0.9	1.0	115	0.9	0.9	0.535	−0.1 [−0.4; 0.2]
Cognition	68	1.3	1.0	115	1.4	1.0	0.733	0.1 [−0.3; 0.4]
Communication	68	1.0	1.0	115	1.2	1.1	0.288	0.2 [−0.1; 0.5]
Bodily discomfort	68	1.9	1.2	115	1.4	1.2	**0.002**	−0.6 [−0.9; −0.2]
Stigma	68	1.2	1.3	115	0.9	1.2	0.090	−0.3 [−0.7; 0.0]
NMSS total (median) [IQR]	68	(57.5)	[37.5; 75.5]	120	(48.5)	[29.5; 86.8]	0.393	−1.3 [−13.0; 5.0]
Cardiovascular	68	(0.0)	[0.0; 2.0]	120	(0.0)	[0.0; 2.0]	0.887	0.0 [0.0; 0.0]
Sleep/fatigue	68	(14.5)	[8.0; 23.5]	120	(15.0)	[8.3; 24.0]	0.989	15.0 [−3.0 ;3.0]
Mood/apathy	68	(4.0)	[1.0; 8.0]	120	(2.5)	[0.0; 10.0]	0.369	3.0 [−2.0; 0.0|
Perceptual problems/hallucinations	68	(0.0)	[0.0; 0.8]	120	(0.0)	[0.0; 1.0]	0.983	0.0 [0.0; 0.0]
Attention/memory	68	(3.0)	[0.3; 8.0]	120	(3.0)	[0.0; 7.8]	0.923	3.0 [−1.0; 1.0]
Gastrointestinal	68	(4.0)	[0.0; 8.0]	120	(4.0)	[0.0; 8.0]	0.894	4.0 [−1.0; 1.0]
Urinary	68	(8.0)	[0.4; 17.0]	120	(6.0)	[2.0; 14.0]	0.294	6.0 [−4.0; 1.0]
Sexual function	68	(0.0)	[0.0; 0.0]	120	(0.0)	[0.0; 6.0]	**0.001**	0.0 [0.0; 0.0]
Miscellaneous	68	(10.0)	[4.3; 17.8]	120	(8.0)	[4.0; 16.0]	0.058	8.0 [−4.0, 0.0]
SCOPA-M total	66	23.3	9.1	117	23.0	7.7	0.827	−0.3 [−2.8; 2.2]
Tremor	67	13.2	15.8	118	18.4	21.2	0.079	5.2 [−0.6; 11.1]
Bradykinesia	67	35.3	20.7	118	39.0	21.0	0.254	3.7 [−2.6, 10.0]
Axial symptoms	67	32.3	18.8	114	28.5	16.2	0.177	−3.7 [−9.2; 1.7]
Dysphagia and dysarthria	67	22.9	16.6	114	23.0	16.2	0.963	0.1 [−4.9; 5.1]
Dyskinesia	66	46.7	29.8	115	33.6	28.8	**0.004**	−13.1 [−22.0; −4.2]
Motor fluctuations	66	47.7	24.1	115	42.8	25.9	0.204	−5.0 [−12.7; 2.7]
*LEDD*	68	1039.0	451.7	121	1146.0	536.1	0.166	107.0 [-44.8; 258.7]

Significant results are highlighted in bold font.

*CI* confidence interval, *IQR* interquartile range, *LEDD* levodopa equivalent daily dose, *n* number, *NMSS* Non-motor Symptom Scale, *PDQ-8 SI* Parkinson’s Disease Questionnaire-8 Summary Index, *SCOPA-M* Scales for Outcomes in Parkinson’s Disease-motor scale.

The PDQ-8 SI ranges from 0 (no impairment) to 100 (maximum impairment). The NMSS total score ranges from 0 (no NMS impairment) to 360 (maximum NMS impairment). SCOPA-M subscores are presented as percentage of maximum domain score. Tremor subscore was based on items 1 and 2; axial subscore on items 5, 6, 7, 9, 15, and 16; bradykinesia subscore on items 3 and 4; dysphagia and dysarthria subscore on items 8, 10, and 11; dyskinesia subscore on items 18 and 19; and ON/OFF fluctuations subscore on items 20 and 21.

### Clinical outcomes of the original cohort

Women and men with PD experienced improvements of the PDQ-8 SI, NMSS total score, and SCOPA-M total score (see Tables 3 and 4) and LEDD reductions. We observed no gender differences in these outcomes at 6-month follow-up in the between-group comparison (all *P* > 0.05). However, post-hoc exploratory analyses of domains of these scores revealed following differences: For the NMSS, beneficial effects were observed in both genders in the ‘sleep/fatigue’, ‘urinary’, and ‘miscellaneous’ domains. Only men with PD significantly improved in ‘mood/apathy’ and ‘perceptual problems/hallucinations’, whereas only women with PD experienced an improvement in ‘attention/memory’. In SCOPA-M subscores, we observed an improvement in both genders for ‘tremor’, ‘dyskinesia’, and ‘motor fluctuations’, whereas ‘bradykinesia’ improved only in men with PD. For the PDQ-8, we observed an improvement in both genders in the ‘mobility’, ‘activities of daily living’, ‘cognition’, ‘bodily discomfort’, and ‘stigma’ domains, whereas ‘emotional well-being’ improved only in men with PD.

**Table 3:** Non-motor and quality of life outcomes at baseline and 6-month follow-up in women and men with Parkinson’s disease.

	Women								Men									
	Baseline			6-MFU			Baseline vs. 6-MFU^a^		Baseline			6-MFU			Baseline vs. 6-MFU^a^		Women vs. men^b^	
	*n*	Mean	SD	*n*	Mean	SD	*P*	∆ [95% CI]	*n*	*Mean*	SD	*n*	*Mean*	SD	*P*	∆ [95% CI]	*P*	∆ [95% CI]
PDQ-8 SI	68	34.3	15.6	67	26.5	15.7	**<0.001**	7.9 [4.1; 11.7]	115	30.5	16.5	117	23.4	16.1	**<0.001**	7.5 [4.5; 10.5]	0.991	−0.4 [−5.2; 4.4]
Mobility	68	1.9	1.0	68	1.5	1.2	**0.049**	0.4 [0.0; 0.7]	115	1.5	1.2	117	1.2	1.1	**0.001**	0.4 [0.1; 0.6]	0.971	0.0 [−0.4; 0.4]
Activities of daily living	68	1.5	1.3	68	1.2	1.2	**0.034**	0.3 [0.0; 0.7]	115	1.5	1.2	117	1.0	1.1	**<0.001**	0.6 [0.3; 0.8]	0.262	0.2 [−0.2; 0.6]
Emotional well-being	68	1.1	0.9	68	1.1	1.0	0.488	0.1 [−0.1; 0.3]	115	1.0	0.9	117	0.8	0.9	**0.048**	0.2 [0.0; 0.4]	0.437	0.1 [−0.2; 0.4]
Social support	68	0.9	1.0	68	0.8	1.0	0.315	0.1 [−0.1; 0.4]	115	0.9	0.9	117	0.8	0.9	0.413	0.1 [−0.1; 0.2]	0.624	−0.1 [−0.4; 0.4]
Cognition	68	1.3	1.0	68	1.0	1.0	**0.010**	0.4 [0.1; 0.6]	115	1.4	1.0	117	1.1	1.0	**0.005**	0.3 [0.1; 0.5]	0.731	−0.1 [−0.4; 0.2]
Communication	68	1.0	1.0	68	0.9	1.1	0.336	0.1 [−0.1; 0.4]	115	1.2	1.1	117	1.0	1.0	0.050	0.2 [0.0; 0.4]	0.736	0.1 [−0.3; 0.4]
Bodily discomfort	68	1.9	1.2	67	1.4	1.2	**0.001**	0.6 [0.3; 0.9]	115	1.4	1.2	117	1.0	1.1	**0.002**	0.4 [0.1; 0.6]	0.294	−0.2 [−0.6;0.2]
Stigma	68	1.2	1.3	68	0.8	1.0	**0.012**	0.4 [0.1; 0.7]	115	0.9	1.2	117	0.6	1.0	**0.002**	0.3 [0.1; 0.5]	0.695	−0.1 [−0.4; 0.3]
NMSS total (median) [IQR]	68	(57.5)	[37.5; 75.5]	68	(37.0)	[24.3; 54.8]	**<0.001**	16.0 [−24.0; −10.5]	120	(48.5)	[29.5; 86.8]	119	(34.0)	[18.0; 54.0]	**<0.001**	15.0 [−22.0; −11.0]	0.991	1.0 [−9.0; 8.0]
Cardiovascular	68	(0.0)	[0.0; 2.0]	68	(0.0)	[0.0; 2.0]	0.435	0.0 [−0.5; 0.0]	120	(0.0)	[0.0; 2.0]	119	(0.0)	[0,0; 1.0]	0.063	0.0 [−0.5; 0.0]	0.456	0.0 [0.0; 0.0]
Sleep/fatigue	68	(14.5)	[8.0; 23.5]	68	(8.0)	[4.0; 14.8]	**<0.001**	5.5 [−8.0; −3.5]	120	(15.0)	[8.3; 24.0]	119	(6.0)	[2.0; 12.0]	**<0.001**	5.5 [−9.0; −5.0]	0.399	−0.5 [−2.0; 4.0]
Mood/apathy	68	(4.0)	[1.0; 8.0]	68	(2.0)	[0.0; 6.8]	0.129	1.0 [−2.5; 0.0]	120	(2.5)	[0.0; 10.0]	119	(1.0)	[0.0; 6.0]	**0.022**	1.0 [−2.5; 0.0]	0.860	1.0 [−1.0; 2.0]
Perceptual problems/ hallucinations	68	(0.0)	[0.0; 0.8]	68	(0.0	[0.0; 0,0]	0.176	0.0 [0.0; 0.0]	120	(0.0)	[0.0; 1.0]	119	(0.0)	[0.0; 0.0]	**0.007**	0.0 [0.0; 0.0]	0.393	0.0 [0.0; 0.0]
Attention/memory	68	(3.0)	[0.3; 8.0]	68	(1.5)	[0.0; 4.0]	**0.008**	1.0 [−3.0; −0.5]	120	(3.0)	[0.0; 7.8]	119	(3.0)	[0.0; 6.0]	0.548	0.0 [−1.0; 0.5]	0.077	1.0 [−3.0; 0.0]
Gastrointestinal	68	(4.0)	[0.0; 8.0]	68	(2.0)	[0.0; 8.0]	0.211	0.0 [−2.0; 0.0]	120	(4.0)	[0.0; 8.0]	119	(2.0)	[0.0; 8.0]	0.338	0.0 [−1.0; 0.0]	0.620	0.0 [−1.0;1.0]
Urinary	68	(8.0)	[0.4; 17.0]	68	(6.0)	[2.0; 12.8]	**0.012**	1.5 [−4.5; −0.5]	120	(6.0)	[2.0; 14.0]	119	(4.0)	[0.0; 11.0]	**0.006**	1.5 [−3.5; −0.5]	0.668	1.5 [−3.0; 2.0]
Sexual function	68	(0.0)	[0.0; 0.0]	68	(0.0)	[0.0; 1.0]	0.525	0.0 [0.0; 0.0]	120	(0.0)	[0.0; 6.0]	119	(0.0)	[0.0; 4.0]	0.128	0.0 [−1.0; 0.0]	0.210	0.0 [0.0; 0.0]
Miscellaneous	68	(10.0)	[4.3; 17.8]	68	(6.0)	[2.3; 12.0]	**<0.001**	4.0 [−6.0; −2.0]	120	(8.0)	[4.0; 16.0]	119	(4.0)	[1.0; 8.0]	**<0.001**	3.0 [−5.0; −2.0]	0.598	1.0 [−3.0; 2.0]

Outcome parameters at baseline and follow-up for women and men with PD. Multiple comparisons due to multiple outcome parameters were corrected with the Benjamini–Hochberg method. Post-hoc, we explored PDQ-8 and NMSS domain outcomes. Significant results are highlighted in bold font.

*6-MFU* 6-month follow-up, *CI* confidence interval, *IQR* interquartile range, *LEDD* levodopa equivalent daily dose, *n* number, *NMSS* Non-motor Symptom Scale, *PDQ-8 SI* Parkinson’s Disease Questionnaire-8 Summary Index.

^a^Dependent sample *t*-tests were used to analyze within-group changes of outcome parameters between baseline and 6-month follow-up.

^b^Independent sample *t*-tests were used to analyze between-group differences of change scores between women and men with PD.

**Table 4:** Motor outcomes and changes in medication requirements at baseline and 6-month follow-up in women and men with Parkinson’s disease.

	Women								Men									
	Baseline			6-MFU			Baseline vs. 6-MFU^a^		Baseline			6-MFU			Baseline vs. 6-MFU^a^		Women vs. men^b^	
	*n*	Mean	SD	*n*	Mean	SD	*P*	∆ [95% CI]	*n*	Mean	SD	*n*	Mean	SD	*P*	∆ [95% CI]	*P*	∆ [95% CI]
SCOPA-M total	66	23.3	9.1	64	17.9	8.2	**<0.001**	5.3 [3.3; 7.3]	117	23.0	7.7	110	15.5	7.4	**<0.001**	7.4 [6.0; 8.8]	0.352	2.1 [−0.3; 4.4]
Tremor	67	13.2	15.8	64	6.3	12.3	**0.002**	6.6 [2.5; 10.8]	118	18.4	21.2	112	13.2	16.9	**0.005**	5.4 [1.7; 9.1]	0.675	−1.2 [−7.0; 4.6]
Bradykinesia	67	35.3	20.7	64	31.9	22.0	0.155	4.2 [−1.6; 9.9]	118	39.0	21.0	112	26.9	17.9	**<0.001**	11.8 [7.3; 16.2]	**0.040**	7.6 [0.4; 14.9]
Axial symptoms	67	32.3	18.8	64	26.9	17.2	**0.013**	5.5 [1.2; 9.7]	114	28.5	16.2	109	20.4	15.1	**<0.001**	7.8 [5.1; 10.6]	0.334	2.4 [−2.4, 7.2]
Dysphagia and dysarthria	67	22.9	16.6	64	20.7	16.5	0.125	2.6 [−0.7; 5.9]	114	23.0	16.2	109	21.1	16.1	0.241	1.9 [−1.1; 5.0]	0.768	−0.7 [5.4; 4.0]
Dyskinesia	66	46.7	29.8	67	26.1	25.2	**<0.001**	20.2 [12.5; 27.9]	115	33.6	28.8	115	16.5	22.9	**<0.001**	16.5 [10.8; 22.2]	0.440	−3.7 [−13.1; 5.7]
Motor fluctuations	66	47.7	24.1	67	29.9	26.4	**<0.001**	17.4 [10.5; 24.3]	115	42.8	25.9	116	20.1	23.2	**<0.001**	22.3 [17.2; 27.5]	0.257	4.9 [−3.6; 13.4]
*LEDD*	68	1039.0	451.7	67	579.4	322.7	**<0.001**	458.7 [348.9; 568.4]	121	1146.0	536.1	121	583.1	336.9	**<0.001**	562.9 [463.5; 662.3]	0.376	104.2 [−51.5; 259.9]

Outcome parameters at baseline and follow-up for women and men with PD. Multiple comparisons due to multiple outcome parameters were corrected with the Benjamini–Hochberg method. Post-hoc, we explored SCOPA-M domain outcomes. Significant results are highlighted in bold font. SCOPA subscores are presented as percentage of maximum domain score. Tremor subscore was based on items 1 and 2; axial subscore on items 5, 6, 7, 9, 15, and 16; bradykinesia subscore on items 3 and 4; dysphagia and dysarthria subscore on items 8, 10, and 11; dyskinesia subscore on items 18 and 19; and ON/OFF fluctuations subscore on items 20 and 21.

*6-MFU* 6-month follow-up, *CI* confidence interval, *IQR* interquartile range, *LEDD* levodopa equivalent daily dose, *n* number, *SCOPA-M* Scales for Outcomes in Parkinson’s Disease-motor scale.

^a^Dependent sample *t*-tests were used to analyze within-group changes of outcome parameters between baseline and 6-month follow-up.

^b^Independent sample *t*-tests were used to analyze between-group differences of change scores between women and men with PD.

The strength of clinical responses for women and men with PD and number needed to treat results are presented in Supplementary Tables 1 and 2. In summary, differences in effect sizes were small and favorable for men in emotional well-being, SCOPA-M total, bradykinesia, motor fluctuations, and LEDD and for women in attention/memory.

### The matched sub-cohort: baseline characteristics and clinical outcomes

Propensity score matching resulted in a sub-cohort of 116 patients (58 women and 58 men). Balance diagnostics indicated a good matching between the two groups with no significant differences for all main demographic and clinical outcome parameters. Baseline characteristics of the matched sub-cohort are reported in Supplementary Table 3 and clinical outcomes in Supplementary Tables 4 and 5. No significant gender differences were observed for all main outcomes at 6-month follow-up. The clinical outcomes of the matched sub-cohort did not differ from the original cohort.

## Discussion

Our study provides evidence of a gender gap in DBS for PD: (1) Disproportionally fewer women underwent DBS indication assessments than to be expected from the gender ratio of the general PD population, (2) preoperatively, mean PD duration was longer and dyskinesia more severe in women with PD, and, (3) nonetheless, DBS was equally clinically efficacious on total QoL, nonmotor, and motor symptoms burden in women and men with PD (Class II evidence).

In our cross-sectional cohort, disproportionally fewer women were referred for DBS indication evaluations as the gender ratio was (men:women) 2.1:1 as compared to the gender proportion in the known PD population 1.48:1^7,14^. In women, 80% of referred patients were approved for DBS, which was significantly higher than the 69% approval rate in men.

The observation that indication evaluations were negative in only 20% of women compared to 31% in men, however does not indicate that the gender effect is beneficial for women with PD as women experienced more severe preoperative motor complications^10^. This bias represents not a local, but a systematic effect and has also been observed in other cohorts in the USA (Miami^8^ and Medicare Services^12^) and Europe (Düsseldorf^15^ and Umeå^16^). Previous studies indicate that the gender gap in assessments of eligibility for DBS may result from nonclinical factors^15,17^ and possible explanations include gender referral biases to specialty care^18,19^, patient preferences regarding medical care^8^ including greater fear of surgery among women^20^, and unmeasured clinical characteristics, such as socioeconomic status^18^. The proportion of rejections due to clinically relevant depression was higher for women with PD. Rejections were based on assessments in multi-disciplinary team meetings in which patients also participated. In these meetings, patients’ history of affective and neurological symptoms, evaluations of expert psychiatrists experienced in DBS indication evaluations for PD, and neuropsychological depression test scores were taken into consideration. As previous studies show that <30% of PD patients consent to referral for DBS evaluations, further research is needed regarding the referral processes of general practitioners and neurologists^21^. Gender ratios in DBS cohorts seem to align better with the PD prevalence when patients receive specially developed educational material and referring medical professionals use DBS screening tools^8,15^. In our study, the odds of undergoing DBS surgery after being evaluated as a good candidate for DBS was ~27% lower in women than in men with PD. To our knowledge, our study is the first to report gender ratios at key steps from referral through indication evaluations until DBS surgery. Further studies are needed to investigate why women with PD approved for DBS treatment do not undergo DBS surgery eventually.

Looking beyond DBS cohorts, women are also underrepresented for invasive treatments in other diseases, such as cardiac^22,23^ or gastrointestinal conditions^24^. Future studies in gender medicine are needed to investigate the deliberation process of patients and the clinical reasoning of referring medical professionals and of hospital staff in which patients undergo invasive treatments. These studies should focus on the decisional process rather than the decision’s end results, which may help to develop new approaches to understand and influence these gender disparities.

In our longitudinal original cohort, in line with previous DBS studies for PD, disease duration was longer in women with PD^8^. This might be explained by the fact that women appear to have slower disease progression^25^. Confirming results of previous studies, women reported more severe motor complications than men before undergoing DBS surgery, which mainly resulted from dyskinesia^16,26^. In line with a study by Hariz et al., we did not observe significant gender differences in total preoperative QoL and women specifically reported worse bodily discomfort and mobility^16^. We observed no preoperative gender differences in overall NMS burden. Confirming previous studies^27^, we observed no gender differences in mood/apathy and attention/memory. Men reported worse preoperative sexual functions than women which is in line with previous research^28^.

In line with previous studies, we observed similar improvements of total QoL and motor outcomes in women and men with PD undergoing DBS in our original cohort^16,29,30^. Our study expanded on the existing literature by systematically comparing gender differences in nonmotor effects of DBS for PD. We observed similar beneficial effects in both genders on total NMS burden. However, we found distinct DBS effect profiles for specific NMS in women and men with PD: Only women experienced an improvement in ‘attention/memory’, whereas only men experienced beneficial effects in the ‘mood/apathy’ and ‘perceptual problems/hallucinations’ domains. Further studies are needed to investigate possible reasons for differential effects on specific NMS in women and men with PD, in particular, considering gender differences in brain structure and function in PD^31^.

In summary, our study presents evidence that different stakeholders may contribute to the gender gap observed in DBS: (1) *general practitioners and neurologists* refer disproportionally fewer women than men for DBS indication evaluations, (2) *women with PD* with positive indication evaluations undergo DBS surgery less likely than men with PD, to which (3) *hospital medical staff* may contribute as all indication assessments are conducted in the setting of in-patient care which provides ample time to convey the rationale and clinical reasoning for a treatment with DBS when indication evaluations are positive. Monitoring gender ratios in DBS is informative, but this does not address the underlying reasons for gender disparities outlined here. Closely connected to this point, the gender gap is still evident despite the implementation of ‘gender mainstreaming strategies’ in healthcare systems around the globe^32^. Therefore, we advocate that more focus should be directed toward the decisional processes and the responsibilities of stakeholders in the implementation of gender equity in the context of DBS treatment. A pioneer interview study included 11 women with PD but lacked clinical data to investigate how patients’ nonmotor or motor symptom profiles influence decision-making processes^33^.

The present work has limitations. In the cross-sectional cohort, the reasons why patients did not receive DBS were analyzed retrospectively and the reasons of DBS referrals were not assessed systematically. Therefore, this study does not consider the number of patients who declined referral for DBS evaluations. Further studies including surveys in referring general practitioners and neurologists are needed. In the longitudinal cohort, although the cohort size of 189 patients was one of the biggest in studies of its kind, especially the group of women with PD (*n* = 68) was relatively small. As this was a “real-world study” we did not examine motor OFF states and all assessments were conducted in clinical ON states. However, NMS and QoL were surveyed over the previous 4 weeks and, therefore, reflect ON and OFF states. Clinical ratings were performed by unblinded raters. However, raters were unaware of the research question regarding gender difference analyses, so a bias resulting from a lack of blinding is improbable. As this was a “real-world study”, we used abbreviated QoL and motor scales (PDQ-8 and SCOPA-motor scale) which highly correlate with the scales from which they were derived (PDQ-39 and UPDRS-III). However, the use of the latter scales may have revealed small differences amongst women and men with PD better due to their finer gradation. Furthermore, minimal clinically important changes have not been published for the NMSS yet. Therefore, the clinical relevance of our results was assessed based on Cohen’s d effect sizes^34^. In our cohort, baseline disease duration was longer and dyskinesia more severe in women with PD. Therefore, we used propensity scores to identify a sub-cohort which was precisely matched for these variables and, thereby, establish a quasi-experimental design to confirm results of the original cohort. While propensity score matching has advantages as a method providing a ‘pseudo-randomization’ in observational studies, it cannot replace a randomized clinical trial. However, in certain scenarios, such as in our database, the real-life presentation of women and men with PD may be of scientific interest. Here, propensity score matching provides an accurate approach to increase causal inference. An inclusion of demographic and clinical parameters in the matching procedure and an implementation of strict comprehensive diagnostic statistics increase the validity of our results. However, this method can only be applied to parameters assessed clinically. Therefore, it should be noted, that there are other contributors to DBS outcomes beyond the factors, which are considered in the propensity score matching, and that this method does not consider potentially relevant parameters, which were not measured, for example impulse control disorders. Acknowledging the possibility of unknown confounders, we used independent samples tests for all further statistical tests for comparisons between women and men with PD^35^. Furthermore, one has to acknowledge that DBS cohorts are highly selected and that dementia and severe depression are considered to be contraindications for DBS. Therefore, our results cannot be generalized to PD patients with severe impairments in these NMS.

Another limitation of this study is the short time period of 6 months for the evaluation of the outcome of DBS in PD patients. However, a similar improvement of both genders was also observed in a study, which analyzed gender differences regarding motor function, dyskinesia, the 36-item Short Form Health Survey, the Mini-Mental State Examination, and the Beck’s Depression Inventory 5 years after STN-DBS^27^. In this study, Kim et al. reported favorable long-term effects of DBS in men on QoL preservation. Further studies are needed to investigate gender differences of nonmotor long-term effects of DBS beyond depression and global cognition.

We observed a gender gap in patients undergoing DBS indication evaluations and treatment. The reasons for this gender gap seem to be nonclinical as the proportion of women with PD with positive indication evaluations who eventually underwent DBS was lower than in men with PD even though DBS efficacy was equal regarding total QoL, nonmotor, and motor symptoms. Therefore, to implement gender equity, we propose that more focus should be spent on nonclinical factors, such as deliberation processes of women with PD and clinical reasoning of referring general practitioners and neurologists. The observation of distinct effect profiles in women and men with PD for specific NMS highlights the need of holistic assessments of nonmotor and motor symptoms in patients with PD undergoing DBS. Therefore, in accordance with the concept of personalized medicine, we advocate considering nonclinical parameters and the evaluation of holistic clinical assessments side-by-side to tailor PD treatment to patients’ individual needs^36^.

## Methods

### Study design and ethical approval

We analyzed longitudinal data from the prospective, observational, multicenter international NILS study^37^ including centers in Cologne, Marburg, Greater Manchester, and London. All patients gave written informed consent before study procedures. The study was performed in accordance with the Declaration of Helsinki (German ClinicalTrials Register DRKS00006735, local ethics committees master votes for Germany: Cologne #12/145, and for UK: NRES South-East London REC3-10/H0808/141 #10084).

In addition, we conducted a retrospective chart review of referrals and indication evaluations for DBS in a cross-sectional cohort of the University Hospital Cologne from January 2015 to September 2020.

### Participants

PD diagnosis was based on the British Brain Bank criteria^38^ in women and men. Patients were screened for DBS according to Movement Disorders Society (MDS) guidelines^39^. DBS surgery was considered when levodopa responsiveness was sufficient (>30% improvement in the Unified PD Rating Scale-motor examination, UPDRS-III). Patients were not eligible for DBS treatment if they suffered from clinically relevant psychiatric diseases or neuropsychological impairments^40^ as assessed by a multidisciplinary team of specialized neurologists, neuropsychologists, stereotactic neurosurgeons, psychiatrists, speech and physiotherapists. In the longitudinal cohort, all patients received bilateral STN-DBS. In the cross-sectional cohort, patients undergoing DBS were implanted in the STN, globus pallidus internus or ventral intermediate nucleus.

### Clinical assessment

Patients were assessed in the ON-medication state (MedON) at preoperative baseline and in a clinical medication and stimulation ON state (MedON/StimON) at postoperative 6-month follow-up. The following scales were assessed:

QoL was assessed with the PD Questionnaire-8 (PDQ-8) which covers eight QoL domains (mobility, activities of daily living, emotional well-being, social support, cognition, communication, bodily discomfort, and stigma)^41^. It is recommended by the MDS Scales Committee for QoL assessments^42^ and has been used in DBS studies^43–46^. The results are presented as PDQ-8 Summary Index (PDQ-8 SI).

NMS were assessed with the NMSScale (NMSS)^47^. The scale consists of 30 items for nine nonmotor domains of PD (cardiovascular, sleep/fatigue, mood/apathy, perceptual problems/hallucinations, attention/memory, gastrointestinal symptoms, urinary symptoms, sexual function, and miscellaneous symptoms).

Motor disorder was assessed with the Scales for Outcomes in PD-motor scale (SCOPA-M). The SCOPA-M is a modified version of the UPDRS^48^, strongly correlates with the corresponding subscales of the UPDRS, and has been used in DBS studies before^37,49,50^. The SCOPA-M was preferred because its assessment time is four times shorter than the MDS-UPDRS^51^. A study by Rooden et al.^52^ combined items from the motor examination and activities of daily living sections of the SCOPA-M and, using a data-driven approach, identified the following motor aspects in an exploratory factor analysis: (1) axial (postural and locomotor) symptoms, (2) axial (general) symptoms, such as speech and swallowing, and ‘freezing during on’, (3) tremor, and (4) bradykinesia and rigidity. As published previously by our group^4^, to better distinguish between these axial symptoms, we included only speech and swallowing in a subscore for ‘dysphagia and dysarthria’ and report subscores for ‘dyskinesia’ and ‘motor fluctuations’.

The levodopa equivalent daily dose (LEDD) was calculated according to the method by Tomlinson et al.^53^.

### Statistical analysis

In the cross-sectional cohort, to analyze clinical practice of referrals and DBS indication assessments for women and men with PD, we conducted a systematic chart review for consecutive patients at the University Hospital Cologne between January 2015 and September 2020. We recorded the total number of referrals and positive and negative indication assessments in women and men with PD. A one-sample binomial test was employed to compare the ratio of women with PD referred for DBS indication to the ratio of women in the general PD population. Further, we analyzed differences in gender proportions using Chi-square tests or Fisher’s exact test, when sample size was low (expected values in any of the cells of a contingency table below 5) at the following key steps: (1) referrals for DBS indication evaluation, (2) positive and negative decisions of indication evaluations, (3) reasons for negative DBS evaluations, and (4) DBS surgery. We calculated relative risk statistics for women compared to men with PD at these steps. The term ‘relative risk’ is used in the statistical sense for the ratio of the probabilities of a certain outcome in two groups, whether the outcome is medically favorable (e.g., treatment with DBS) or unfavorable (e.g., development of complications). To compare the total relative risk for DBS treatment of women compared to men with PD, we multiplied the relative risks of referral with those of following steps (positive indication evaluations and DBS surgery).

In the longitudinal cohort, we analyzed clinical outcomes of women and men with PD. Normal distribution was tested with the Shapiro–Wilk test. Preoperative gender differences were analyzed using Mann–Whitney *U* tests or unpaired *t*-tests when parametric test criteria were fulfilled. Within-group changes at 6-month follow-up were analyzed with Wilcoxon signed-rank or paired samples *t*-tests, when parametric test criteria were fulfilled. Change scores (test_baseline_ − test_follow-up_) were calculated to compare differences between men and women using Mann–Whitney *U* or unpaired *t*-tests. In addition, we computed relative changes ([test_baseline_ − test_follow-up_]/test_baseline_) and Cohen’s effect sizes including confidence intervals based on non-central t distribution according to a method by Smithson^54^. Furthermore, we calculated number needed to treat (1/% of patients improving > ½ SD test_baseline pooled_). Multiple comparisons due to multiple outcome parameters were corrected with the Benjamini–Hochberg method. All *P* values presented are adjusted to the *P* < 0.05 significance threshold. Post-hoc, we explored PDQ-8, NMSS, and SCOPA-M domain outcomes. In addition, to account for potential baseline differences between women and men with PD, we used Propensity Score Matching for SPSS (version 3.04)^55^. Matching variables were age at intervention, disease duration, and baseline SCOPA-dyskinesia. We implemented nearest-neighbor matching with a 0.25 caliper^56^ without replacement employing a 1:1 ratio (women:men). Balance diagnostics were conducted based on Cohen’s effect size |d | <0.25^56^. Subsequently, all analyses of clinical changes were also carried out for the thus identified matched sub-cohort.

### Reporting summary

Further information on research design is available in the Nature Research Reporting Summary linked to this article.

## Supplementary information

Supplementary document

Reporting Summary

## Data Availability

The data used to support the findings of this study are available from the corresponding authors upon reasonable request.

## Code availability

Statistical analyses were performed using SPSS Statistics 25. SPSS codes are available upon reasonable request to the corresponding authors.
