# Supplementary material for: Gender gap in deep brain stimulation for Parkinson’s disease
Source: NPJ Parkinsons Dis. 2022 Apr 20;8:47. doi: 10.1038/s41531-022-00305-y (PMC9021281; doi:10.1038/s41531-022-00305-y)
Supplement: Supplementary file 1 — Supplementary document [file 41531_2022_305_MOESM1_ESM.docx]

**Supplementary Table 1 – Change scores, relative changes and effect sizes for women and men with PD.**

|  | **Change score** | |  | **Relative Change (%)** | |  | **Effect size** | |
| --- | --- | --- | --- | --- | --- | --- | --- | --- |
|  | **Women** | **Men** |  | **Women** | **Men** |  | **Cohen‘s *d***  [95% *CI*] | **Classification of difference** |
| **PDQ-8 SI** | 7.8 | 7.1 |  | 22.7 | 23.3 |  | 0.02 [-0.3; 0.3] | No effect |
| Mobility | 0.4 | 0.3 |  | 21.1 | 20.0 |  | 0.01 [-0.2; 0.2] | No effect |
| Activities of daily living | 0.3 | 0.5 |  | 20.0 | 33.3 |  | 0.17 [-0.1; 0.5] | No effect |
| Emotional well-being | 0.0 | 0.2 |  | 0.0 | 20.0 |  | 0.12 [-0.2; 0.4] | Small effect favoring men |
| Social support | 0.1 | 0.1 |  | 11.1 | 11.1 |  | 0.08 [-0.2; 0.4] | No effect |
| Cognition | 0.3 | 0.3 |  | 23.1 | 21.4 |  | 0.05 [-0.2; 0.4] | No effect |
| Communication | 0.1 | 0.2 |  | 10.0 | 16.7 |  | -0.05 [-0.2; 0.4] | No effect |
| Bodily discomfort | 0.5 | 0.4 |  | 26.3 | 28.6 |  | 0.16 [-0.1; 0.5] | No effect |
| Stigma | 0.4 | 0.3 |  | 33.3 | 33.3 |  | 0.06 [-0.2; 0.4] | No effect |
| **NMSS total** | 19.4 | 19.2 |  | 32.0 | 32.4 |  | 0.00 [-0.1; 0.1] | No effect |
| Cardiovascular | 0.7 | 0.5 |  | 36.8 | 33.3 |  | 0.03 [-0.3; 0.3] | No effect |
| Sleep/fatigue | 6.6 | 8.0 |  | 40.5 | 49.1 |  | 0.13 [-0.2; 0.4] | No effect |
| Mood/apathy | 1.7 | 2.3 |  | 26.2 | 31.5 |  | 0.05 [-0.2; 0.4] | No effect |
| Perceptual problems/ hallucinations | 0.4 | 0.8 |  | 28.6 | 61.5 |  | 0.13 [-0.2; 0.4] | No effect |
| Attention/memory | 1.6 | 0.2 |  | 33.3 | 4.1 |  | 0.24 [-0.1; 0.5] | Small effect favoring women |
| Gastrointestinal | 1.1 | 0.7 |  | 20.0 | 13.0 |  | 0.07 [-0.2; 0.4] | No effect |
| Urinary | 3.1 | 2.5 |  | 29.2 | 27.2 |  | 0.07 [-0.2; 0.4] | No effect |
| Sexual function | -0.3 | 0.6 |  | -27.3 | 16.7 |  | 0.21 [-0.1; 0.5] | No effect |
| Miscellaneous | 4.6 | 3.8 |  | 36.2 | 38.0 |  | 0.08 [-0.2; 0.4] | No effect |
| **SCOPA-M total** | 5.4 | 7.5 |  | 23.2 | 32.6 |  | 0.27 [0.0; 0.6] | Small effect favoring men |
| Tremor | 6.9 | 5.2 |  | 52.3 | 28.3 |  | 0.07 [-0.2; 0.4] | No Effect |
| Bradykinesia | 3.4 | 12.1 |  | 9.6 | 31.0 |  | 0.32 [0.0; 0.6] | Small effect favoring men |
| Axial symptoms | 5.4 | 8.1 |  | 16.7 | 28.4 |  | 0.15 [-0.2; 0.5] | No effect |
| Dysphagia and dysarthria | 2.2 | 1.9 |  | 9.6 | 8.3 |  | 0.05 [-0.3; 0.4] | No effect |
| Dyskinesia | 20.6 | 17.1 |  | 44.1 | 50.9 |  | 0.12 [-0.2; 0.4] | No effect |
| Motor fluctuations | 17.8 | 22.7 |  | 37.3 | 53.0 |  | 0.18 [-0.1; 0.5] | No effect |
| **LEDD** | 459.6 | 562.9 |  | 44.2 | 49.1 |  | 0.20 [-0.1; 0.5] | Small effect favoring men |

**Legend:** Effect size: small 0.20-0.49, moderate 0.50-0.79, and large >0.80

Change score = (mean test_baseline_ – mean test_follow-up_)

Relative change = (mean test_baseline_ – mean test_follow-up_) / mean test_baseline_ x 100.

Cohen’s d calculated according to a method by Smithson^28^

**Abbreviations: CI** = confidence interval; **NMSS** = Non-motor Symptom Scale; **PD =** Parkinson’s disease; **PDQ-8 SI** = Parkinson’s Disease Questionnaire-8 Summary Index; **SCOPA-M**= Scales for Outcomes in Parkinson’s Disease-motor scale

# Supplementary Table 2 – Number needed to treat.

|  |  | **Number needed to treat** | |
| --- | --- | --- | --- |
|  |  | Women | Men |
| **PDQ-8 SI** |  | 1.9 | 2.1 |
| Mobility |  | 2.3 | 2.5 |
| Activities of daily living |  | 2.3 | 2.2 |
| Emotional well-being |  | 4.3 | 2.9 |
| Social support |  | 3.8 | 3.5 |
| Cognition |  | 2.6 | 2.2 |
| Communication |  | 3.2 | 3.3 |
| Bodily discomfort |  | 1.9 | 2.3 |
| Stigma |  | 2.5 | 3.7 |
| **NMSS total** |  | 2.1 | 2.2 |
| Cardiovascular |  | 6.2 | 4.6 |
| Sleep/fatigue |  | 2.0 | 1.9 |
| Mood/apathy |  | 4.5 | 4.2 |
| Perceptual problems/ hallucinations |  | 7.6 | 6.9 |
| Attention/memory |  | 2.6 | 3.8 |
| Gastrointestinal |  | 4.3 | 3.8 |
| Urinary |  | 3.1 | 3.5 |
| Sexual function |  | 9.7 | 4.7 |
| Miscellaneous |  | 2.3 | 2.6 |
| **SCOPA-M total** |  | 1.8 | 1.5 |
| Tremor |  | 3.4 | 3.1 |
| Bradykinesia |  | 3.2 | 2.0 |
| Axial symptoms |  | 2.4 | 2.0 |
| Dysphagia and dysarthria |  | 2.6 | 2.5 |
| Dyskinesia |  | 1.7 | 2.0 |
| Motor fluctuations |  | 2.1 | 2.0 |
| **LEDD** |  | 1.5 | 1.5 |

**Legend:**

Number needed to treat = (1 / % of patients who improved > ½ SD_baseline pooled_) x 100

**Abbreviations: LEDD** = levodopa equivalent daily dose; **NMSS** = Non-motor Symptom Scale; **PDQ-8** **SI** = Parkinson’s Disease Questionnaire-8 Summary Index; **SCOPA-M** = Scales for Outcomes in Parkinson’s Disease-motor scale.

**Supplementary Table 3 – Baseline characteristics of women and men with PD in the matched sub-cohort.**

|  | **Women** | | | | **Men** | | | | **Women vs. Men** | |
| --- | --- | --- | --- | --- | --- | --- | --- | --- | --- | --- |
|  | *n* | *mean* | *SD* |  | *n* | *mean* | *SD* |  | *P* | ∆ [95% *CI*] |
| **Age** | 58 | 62.3 | 8.5 |  | 58 | 62.2 | 8.5 |  | 0.929 | 0.1 [-3.0; 3.3] |
| **Disease duration** | 58 | 11.3 | 4.8 |  | 58 | 10.9 | 4.4 |  | 0.651 | 0.4 [-1.3; 2.1] |
| **PDQ-8 SI** | 58 | 34.1 | 16.3 |  | 57 | 32.3 | 18.5 |  | 0.578 | 1.8 [-4.6; 8.3] |
| Mobility | 58 | 1.8 | 1.1 |  | 57 | 1.5 | 1.3 |  | 0.154 | 0.3 [-0.1; 0.8] |
| Activities of daily living | 58 | 1.4 | 1.2 |  | 57 | 1.5 | 1.2 |  | 0.452 | 0.3 [-0.6; 0.3] |
| Emotional well-being | 58 | 1.0 | 0.9 |  | 57 | 1.0 | 1.0 |  | 0.689 | 0.1 [-0.3; 0.4] |
| Social support | 58 | 1.0 | 1.0 |  | 57 | 0.9 | 1.0 |  | 0.921 | 0.0 [-0.4; 0.4] |
| Cognition | 58 | 1.4 | 1.1 |  | 57 | 1.4 | 1.1 |  | 0.958 | 0.0 [-0.4; 0.4] |
| Communication | 58 | 1.0 | 1.0 |  | 57 | 1.4 | 1.1 |  | 0.081 | 0.4 [0.0; 0.7] |
| Bodily discomfort | 58 | 2.0 | 1.1 |  | 57 | 1.6 | 1.3 |  | 0.051 | 0.4 [0.0; 0.9] |
| Stigma | 58 | 1.3 | 1.3 |  | 57 | 1.0 | 1.3 |  | 0.226 | 0.3 [-0.2; 0.8] |
| **NMSS total (median) [IQR]** | 58 | (57.0) | [36.5; 74.5] |  | 58 | (44.0) | [28.8; 89.3] |  | 0.414 | -50.0 [-7.0: 16.0] |
| Cardiovascular | 58 | (0.0) | [0.0; 3.3] |  | 58 | (0.0) | [0.0; 1.0] |  | 0.183 | 0.0 [0.0; 0.0] |
| Sleep/fatigue | 58 | (14.5) | [8.0; 22.5] |  | 58 | (14.5) | [7.8; 24.3] |  | 0.936 | -14.5 [-4.0; 4.0] |
| Mood/apathy | 58 | (3.0) | [1.0; 8.0] |  | 58 | (3.5) | [0.0; 10.5] |  | 0.796 | -3.0 [-2.0; 1.0] |
| Perceptual  problems/hallucinations | 58 | (0.0) | [0.0; 1.0] |  | 58 | (0.0) | [0.0; 0.0] |  | 0.539 | 0.0 [0.0; 0.0] |
| Attention/memory | 58 | (3.5) | [0.8; 8.0] |  | 58 | (3.0) | [0.0; 7.3] |  | 0.535 | -3.0 [-1.0; 2.0] |
| Gastrointestinal | 58 | (2.5) | [0.0; 8.0] |  | 58 | (3.0) | [0.0; 6.5] |  | 0.555 | -3.0 [-1.0; 1.0] |
| Urinary | 58 | (6.5) | [3.8; 16.3] |  | 58 | (4.0) | [1.0; 12.0] |  | 0.086 | -5.5 [0.0; 4.0] |
| Sexual function | 58 | (0.0) | [0.0; 0.0] |  | 58 | (0.0) | [0.0; 6.5] |  | **0.002** | **0.0 [-1.0; 0.0]** |
| Miscellaneous | 58 | (9.0) | [4.0; 17.0] |  | 58 | (9.5) | [4.0; 17.0] |  | 0.605 | -9,5 [-2.0; 4.0] |
| **SCOPA-M total** | 58 | 22.7 | 8.6 |  | 58 | 24.1 | 7.9 |  | 0.370 | -1.4 [-4.4; 1.7] |
| Tremor | 58 | 12.8 | 16.1 |  | 58 | 19.0 | 20.0 |  | 0.070 | -6.2 [-12.9; 0.5] |
| Bradykinesia | 58 | 34.6 | 20.6 |  | 58 | 37.2 | 21.3 |  | 0.507 | -2.6 [-10.3; 5.1] |
| Axial symptoms | 58 | 31.6 | 19.1 |  | 58 | 30.3 | 16.1 |  | 0.683 | 1.3 [-5.2; 7.8] |
| Dysphagia and dysarthria | 58 | 22.6 | 16.8 |  | 58 | 22.6 | 17.8 |  | 1.0 | 0.0 [-6.35; 6.35] |
| Dyskinesia | 58 | 46.0 | 27.5 |  | 58 | 45.1 | 28.3 |  | 0.868 | 0.9 [-9.4; 11.1] |
| Motor fluctuations | 58 | 46.6 | 23.9 |  | 58 | 49.4 | 21.4 |  | 0.497 | -2.9 [-11.2;5.5] |
| **LEDD** | 58 | 1059.2 | 463.1 |  | 58 | 1155.4 | 493.5 |  | 0.281 | -96.2 [-272.3; 79.8] |

**Legend:** Significant results are highlighted in bold font. SCOPA-M subscores are presented as percentage of maximum domain score. Tremor subscore was based on items 1 and 2; axial subscore on items 5, 6, 7, 9, 15, and 16; bradykinesia subscore on items 3 and 4; dysphagia and dysarthria subscore on items 8, 10, and 11; dyskinesia subscore on items 18 and 19; and ON/OFF fluctuations subscore on items 20 and 21.

**Abbreviations: CI** = confidence interval; **IQR** = interquartile range; **LEDD** = levodopa equivalent daily dose; **n** = number; **NMSS** = Non-motor Symptom Scale; **PD =** Parkinson’s disease; **PDQ-8** **SI** = Parkinson’s Disease Questionnaire-8 Summary Index; **SCOPA-M** = Scales for Outcomes in Parkinson’s Disease-motor scale

**Supplementary Table 4 – Non-motor and quality of life outcomes at baseline and 6-month follow-up in women and men with PD in the matched sub-cohort.**

|  | **Women** | | | | | | | | |  | **Men** | | | | | | | | | |  | |  | |  |
| --- | --- | --- | --- | --- | --- | --- | --- | --- | --- | --- | --- | --- | --- | --- | --- | --- | --- | --- | --- | --- | --- | --- | --- | --- | --- |
|  | **Baseline** | | |  | **6-MFU** | | | **Baseline vs 6-MFU*** | |  | **Baseline** | | |  | **6-MFU** | | | **Baseline vs 6-MFU*** | | |  | | **Men vs women^†^** | | |
|  | *n* | *mean* | *SD* |  | *n* | *mean* | *SD* | *P* | ∆ [95% *CI*] |  | *n* | *mean* | *SD* |  | *n* | *mean* | *SD* | *P* | ∆ [95% *CI*] |  | | *P* | | ∆ [95% *CI*] | |
| **PDQ-8 SI** | 58 | 34.1 | 16.3 |  | 57 | 26.9 | 15.4 | **<0.001** | 7.4 [3.5; 11.2] |  | 57 | 32.3 | 18.5 |  | 56 | 24.9 | 15.5 | **0.003** | 7.6 [2.6; 12.5] |  | | 0.948 | | -0.2 [-6.4; 5.9] | |
| Mobility | 58 | 1.8 | 1.1 |  | 58 | 1.5 | 1.2 | 0.127 | 0.3 [-0.1; 0.7] |  | 57 | 1.5 | 1.3 |  | 56 | 1.3 | 1.1 | **0.043** | 0.3 [0.0; 0.6] |  | | 0.996 | | 0.0 [-0.5; 0.5] | |
| Activities of daily living | 58 | 1.4 | 1.2 |  | 58 | 1.1 | 1.2 | 0.155 | 0.2 [-0.1; 0.6] |  | 57 | 1.5 | 1.2 |  | 56 | 1.0 | 1.1 | 0.007 | 0.5 [0.1; 0.8] |  | | 0.362 | | -0.2 [-0.7; 0.2] | |
| Emotional well-being | 58 | 1.0 | 0.9 |  | 58 | 1.1 | 0.9 | 0.874 | 0.0 [-0.2; 0.2] |  | 57 | 1.0 | 1.0 |  | 56 | 0.8 | 0.9 | 0.261 | 0.2 [-0.1; 0.5] |  | | 0.318 | | -0.2 [-0.5; 0.2] | |
| Social support | 58 | 1.0 | 1.0 |  | 58 | 0.8 | 0.9 | 0.192 | 0.2 [-0.1; 0.4] |  | 57 | 0.9 | 1.0 |  | 56 | 0.8 | 0.8 | 0.478 | 0.1 [-0.2; 0.4] |  | | 0.752 | | 0.1 [-0.3; 0.5] | |
| Cognition | 58 | 1.4 | 1.1 |  | 58 | 1.0 | 1.0 | **0.011** | 0.4 [0.1; 0.7] |  | 57 | 1.4 | 1.1 |  | 56 | 1.1 | 1.0 | 0.137 | 0.3 [-0.1; 0.6] |  | | 0.556 | | 0.1 [-0.3; 0.6] | |
| Communication | 58 | 1.0 | 1.0 |  | 58 | 1.0 | 1.1 | 0.799 | 0.0 [-0.2; 0.3] |  | 57 | 1.4 | 1.1 |  | 56 | 1.1 | 1.0 | 0.075 | 0.3 [0.0; 0.6] |  | | 0.239 | | -0.2 [-0.6; 0.2] | |
| Bodily discomfort | 58 | 2.0 | 1.1 |  | 57 | 1.5 | 1.2 | **0.002** | 0.6 [0.2; 0.9] |  | 57 | 1.6 | 1.3 |  | 56 | 1.2 | 1.2 | **0.040** | 0.4 [0.0; 0.7] |  | | 0.421 | | 0.2 [-0.3; 0.7] | |
| Stigma | 58 | 1.3 | 1.3 |  | 58 | 0.9 | 1.0 | **0.012** | 0.4 [0.1; 0.7] |  | 57 | 1.0 | 1.3 |  | 56 | 0.7 | 1.0 | **0.023** | 0.3 [0.1; 0.6] |  | | 0.754 | | 0.1 [-0.4; 0.5] | |
| **NMSS total (median) [IQR]** | 58 | (57.0) | [36.5; 74.5] |  | 58 | 39.0 | 31.3 | **<0.001** | 11.0  [-21.0; -7.0] |  | 58 | (44.0) | [28.8; 89.3] |  | 57 | 34.0 | 37.0 | **<0.001** | 17.0  [-24.0; -10.0] |  | | 0.483 | | 6.0  [-13.0; 7.0] | |
| Cardiovascular | 58 | (0.0) | [0.0; 3.3] |  | 58 | (0.0) | [0.0; 2.0] | 0.372 | 0.0 [0.0; 0.0] |  | 58 | (0.0) | [0.0; 1.0] |  | 57 | (0.0) | [0.0; 1.0] | 0.787 | 0.0 [0.0; 0.0] |  | | 0.917 | | 0.0 [0.0; 0,0] | |
| Sleep/fatigue | 58 | (14.5) | [8.0; 22.5] |  | 58 | (8.5) | [4.0; 15.3] | **<0.001** | 4.0 [-7.5; -3.0] |  | 58 | (14.5) | [7.8; 24.3] |  | 57 | (7.0) | [3.0; 12.0] | **<0.001** | 5.0 [-9.5; -4.0] |  | | 0.512 | | 1.0 [-5.0; 2.0] | |
| Mood/apathy | 58 | (3.0) | [1.0; 8.0] |  | 58 | (2.5) | [0.0; 8.0] | 0.561 | 0.0 [-2.0; 1.0] |  | 58 | (3.5) | [0.0; 10.5] |  | 57 | (1.0) | [1.0; 6.0] | **0.027** | 1.0 [-3.5; 0.0] |  | | 0.228 | | 1.0 [-3.0; 1.0] | |
| Perceptual problems/  hallucinations | 58 | (0.0) | [0.0; 1.0] |  | 58 | (0.0) | [0.0; 0.0] | 0.280 | 0.0 [0.0;0.0] |  | 58 | (0.0) | [0.0; 0.0] |  | 57 | (0.0) | [0.0; 0.0] | 0.099 | 0.0 [0.0; 0.0] |  | | 0.489 | | 0.0 [0.0; 0.0] | |
| Attention/memory | 58 | (3.5) | [0.8; 8.0] |  | 58 | (2.0) | [0.0; 4.0] | **0.013** | 1.0 [-3.5; -0.5] |  | 58 | (3.0) | [0.0; 7.3] |  | 57 | (3.0) | [0.0; 6.0] | 0.756 | 0.0 [-1.5; 1.0] |  | | 0.079 | | -1.0 [0.0; 4.0] | |
| Gastrointestinal | 58 | (2.5) | [0.0; 8.0] |  | 58 | (2.0) | [0.0; 8.0] | 0.521 | 0.0 [-1.5; 0.5] |  | 58 | (3.0) | [0.0; 6.5] |  | 57 | (2.0) | [0.0; 7.5] | 0.819 | 0.0 [-2.0; 1.0] |  | | 0.691 | | 0.0 [-1.0; 1.0] | |
| Urinary | 58 | (6.5) | [3.8; 16.3] |  | 58 | (6.0) | [2.0; 12.3] | **0.021** | 1.0 [-4.5; 0.0] |  | 58 | (4.0) | [1.0; 12.0] |  | 57 | (4.0) | [0.0; 8.0] | 0.150 | 0.0 [-3.0; 0.5] |  | | 0.506 | | -1.0 [-2.0; 3.0] | |
| Sexual function | 58 | (0.0) | [0.0; 0.0] |  | 58 | (0.0) | [0.0; 2.0] | 0.464 | 0.0 [0.0; 0.0] |  | 58 | (0.0) | [0.0; 6.5] |  | 57 | (0.0) | [0.0; 4.0] | 0.080 | 0.0 [-2.0; 0.0] |  | | 0.087 | | 0.0 [-1.0; 0.0] | |
| Miscellaneous | 58 | (9.0) | [4.0; 17.0] |  | 58 | (6.0) | [2.0; 12.3] | **0.012** | 3.5 [-6.0; -0.5] |  | 58 | (9.5) | [4.0; 17.0] |  | 57 | (5.0) | [2.0; 10.0] | **<0.001** | 3.0 [-7.0; -2.0] |  | | 0.579 | | -0.5 [-4.0; 2.0] | |

Legend: Outcome parameters at baseline and follow-up for women and men with PD. Multiple comparisons due to multiple outcome parameters were corrected with the Benjamini-Hochberg method. Post-hoc, we explored PDQ-8 and NMSS, domain outcomes. Significant results are highlighted in bold font.

* Wilcoxon signed-rank or paired samples *t*-tests were used to analyze within-group changes of outcome parameters between baseline and 6-month follow-up.

^†^ Mann-Whitney *U* or unpaired *t*-tests were used to analyze between-group differences of change scores between women and men with PD.

Significant results are highlighted in bold font.

**Abbreviations: 6-MFU** = 6-month follow-up; **CI** = confidence interval; **IQR** = interquartile range; **LEDD** = levodopa equivalent daily dose; **n** = number; **NMSS** = Non-motor Symptom Scale; **PD =** Parkinson’s disease; **PDQ-8** **SI** = Parkinson’s Disease Questionnaire-8 Summary Index.

**Supplementary Table 5 – Motor outcomes and changes in medication requirements at baseline and 6-month follow-up in women and men with PD in the matched sub-cohort.**

|  | **Women** | | | | | | | | |  | **Men** | | | | | | | | |  |  | | |
| --- | --- | --- | --- | --- | --- | --- | --- | --- | --- | --- | --- | --- | --- | --- | --- | --- | --- | --- | --- | --- | --- | --- | --- |
|  | **Baseline** | | |  | **6-MFU** | | | **Baseline vs 6-MFU*** | |  | **Baseline** | | |  | **6-MFU** | | | **Baseline vs 6-MFU*** | |  | **Men vs women^†^** | |  |
|  | *n* | *mean* | *SD* |  | *n* | *mean* | *SD* | *P* | ∆ [95% *CI*] |  | *n* | *mean* | *SD* |  | *n* | *mean* | *SD* | *P* | ∆ [95% *CI*] |  | *P* | ∆ [95% *CI*] |  |
| **SCOPA-M total** | 58 | 22.7 | 8.6 |  | 55 | 17.4 | 7.9 | **<0.001** | 5.5 [3.3; 7.6] |  | 58 | 24.1 | 7.9 |  | 55 | 16.1 | 7.6 | **<0.001** | 7.8 [5.7; 9.9] |  | 0.127 | -2.3 [-5.3; 0.7] |  |
| Tremor | 58 | 12.8 | 16.1 |  | 55 | 5.3 | 11.5 | **0.003** | 7.1 [2.5; 11.8] |  | 58 | 19.0 | 20.0 |  | 55 | 12.3 | 17.2 | **0.002** | 7.1 [2.6; 11.7] |  | 1.0 | 0.0 [-6.4; 6.4] |  |
| Bradykinesia | 58 | 34.6 | 20.6 |  | 55 | 32.0 | 21.6 | 0.283 | 3.5 [-3.0; 9.9] |  | 58 | 37.2 | 21.3 |  | 55 | 27.3 | 18.7 | **0.014** | 8.8 [1.8; 15.7] |  | 0.265 | - 5.3 [-14.7; 4.1] |  |
| Axial symptoms | 58 | 31.6 | 19.1 |  | 55 | 26.0 | 16.8 | **0.013** | 5.8 [1.3; 10.2] |  | 58 | 30.3 | 16.1 |  | 54 | 21.4 | 14.2 | **<0.001** | 8.8 [4.7; 13.0] |  | 0.312 | -3.1 [-9.1; 2.9] |  |
| Dysphagia and dysarthria | 58 | 22.6 | 16.8 |  | 55 | 19.4 | 16.2 | **0.038** | 3.6 [0.2; 7.1] |  | 58 | 22.6 | 17.8 |  | 54 | 22.4 | 16.4 | 0.786 | -0.6 [-5.2; 3.9] |  | 0.136 | 4.3 [-1.4; 9.9] |  |
| Dyskinesia | 58 | 46.0 | 27.5 |  | 58 | 24.1 | 25.4 | **<0.001** | 21.8 [14.4; 29.2] |  | 58 | 45.1 | 28.3 |  | 56 | 19.6 | 24.8 | **<0.001** | 25.3 [16.3; 34.3] |  | 0.552 | -3.5 [-14.9; 8.0] |  |
| Motor fluctuations | 58 | 46.6 | 23.9 |  | 58 | 30.7 | 25.9 | **<0.001** | 15.8 [8.8; 22.8] |  | 58 | 49.4 | 21.4 |  | 57 | 25.7 | 24.2 | **<0.001** | 24.3 [17.2; 31.3] |  | 0.091 | -8.5 [-18.3; 1.4] |  |
| **LEDD** | 58 | 1059.2 | 463.1 |  | 58 | 573.9 | 313.0 | **<0.001** | 485.3  [369.7; 600.8] |  | 58 | 1155.4 | 493.5 |  | 58 | 577.1 | 301.9 | **<0.001** | 578.3  [445.7; 710.8] |  | 0.292 | -93.0  [-266.9; 80.9] |  |

Legend: Outcome parameters at baseline and follow-up for women and men with PD. Multiple comparisons due to multiple outcome parameters were corrected with the Benjamini-Hochberg method. Post-hoc, we explored SCOPA domain outcomes. Significant results are highlighted in bold font. SCOPA-M subscores are presented as percentage of maximum domain score. Tremor subscore was based on items 1 and 2; axial subscore on items 5, 6, 7, 9, 15, and 16; bradykinesia subscore on items 3 and 4; dysphagia and dysarthria subscore on items 8, 10, and 11; dyskinesia subscore on items 18 and 19; and ON/OFF fluctuations subscore on items 20 and 21.

* Wilcoxon signed-rank or paired samples *t*-tests were used to analyze within-group changes of outcome parameters between baseline and 6-month follow-up.

^†^ Mann-Whitney *U* or unpaired *t*-tests were used to analyze between-group differences of change scores between women and men with PD.

Significant results are highlighted in bold font.

**Abbreviations: 6-MFU** = 6-month follow-up; **CI** = confidence interval; **IQR** = interquartile range; **LEDD** = levodopa equivalent daily dose; **n** = number; **PD =** Parkinson’s disease; **SCOPA-M** = Scales for Outcomes in Parkinson’s Disease-motor scale.

# Non-Motor Parkinson's Disease Study Group

Members of the MDS Non-Motor Parkinson's Disease Study Group listed here did not contribute to the current study.

Adler, Charles^15^

Bhidayasiri, Roongroj^16^

Borghammer, Per^17^
Barone, Paolo^18^
Brooks, David J.^19^
Brown, Richard^20^
Cantillon, Marc^21^
Carroll, Camille^22^
Coelho, Miguel^23^

Falup-Pecurariu, Cristian^24^
Henriksen, Tove^25^
Hu, Michele^26^

Jenner, Peter^27^

Jeon, Beomseok^28^
Kramberger, Milica^29^
Kumar, Padma^30^
Kurtis, Mónica^31^

Leta, Valentina^9^
Lewis, Simon^32^
Litvan, Irene^33^
Lyons, Kelly^34^
Martino, Davide^35^
Masellis, Mario^36^
Mochizuki, Hideki^37^
Morley, James F.^38^
Nirenberg, Melissa^39^

Odin, Per^40^
Pagonabarraga, Javier^41^
Panicker, Jalesh^42^
Pavese, Nicola^43^
Pekkonen, Eero^44^
Postuma, Ron^45^

Rodriguez Violante, Mayela^46^
Rosales, Raymond^47^
Schapira, Anthony^48^

Schrag, Anette^49^
Simuni, Tanya^50^
Stocchi, Fabrizio^51^

Storch, Alexander^52^
Subramanian, Indu^53^
Tagliati, Michele^54^
Tinazzi, Michele^55^
Toledo, Jon^56^
Tsuboi, Yoshio^57^
Walker, Richard^58^

Weintraub, Daniel^59^

^15^ The Parkinson's Disease and Movement Disorders Center, Department of Neurology, Mayo Clinic, Scottsdale, Arizona, USA

^16^Chulalongkorn Centre of Excellence for Parkinson's Disease & Related Disorders, Department of Medicine, Faculty of Medicine, Chulalongkorn University and King Chulalongkorn Memorial Hospital, Thai Red Cross Society, Bangkok, Thailand

^17^ Nuclear Medicine and PET, Aarhus University Hospital, Aarhus, Denmark

^18^ Center for Neurodegenerative Diseases (CEMAND), Neuroscience Section, University of Salerno, Salerno, Italy

^19^ Institute of Neuroscience, Newcastle University, Newcastle, UK; Department of Nuclear Medicine and PET Centre, Aarhus University Hospital, Aarhus, Denmark

^20^ King's College London, Department of Psychology, London, UK

^21^ Reviva Pharmaceuticals, Inc., Santa Clara, CA, USA

^22^ Faculty of Medicine and Dentistry, University of Plymouth, Plymouth, UK

^23^ FAS Center for Systems Biology, Harvard University, Cambridge, MA, USA

^24^ Faculty of Medicine, Transilvania University of Brașov, Brașov, Romania

^25^ Movement Disorder Clinic, University Hospital of Bispebjerg, Copenhagen, NV, Denmark

^26^ Oxford Parkinson's Disease Centre, University of Oxford, UK; Nuffield Department of Clinical Neurosciences, University of Oxford, UK

^27^ Neurodegenerative Diseases Research Group, Institute of Pharmaceutical Sciences, Faculty of Life Sciences and Medicine, King's College London, Newcomen Street, London, UK

^28^ Department of Neurology, Seoul National University College of Medicine, Seoul, South Korea

^29^ Division of Clinical Geriatrics, Department of Neurobiology, Care Sciences and Society, Center for Alzheimer Research, Karolinska Institutet, Stockholm, Sweden Department of Neurology, University Medical Centre Ljubljana, Ljubljana, Slovenia

^30^ Parkinson's Disease Service for the Older Person, Rankin Park Centre, John Hunter Hospital, HNELHD, Newcastle, NSW, Australia

^31^ Functional Movement Disorders Unit, Movement Disorders Program, Neurology Department, Hospital Ruber Internacional, Madrid, Spain

^32^ Brain and Mind Centre, University of Sydney, NSW, Australia

^33^ Department of Neurosciences Movement Disorders Center, University of California, San Diego, USA

^34^ University of Kansas Medical Center, Kansas City, KS, USA

^35^ Department of Clinical Neurosciences, University of Calgary & Hotchkiss Brain Institute, Calgary, Canada

^36^ Hurvitz Brain Sciences Program, Sunnybrook Research Institute, Toronto, ON, Canada

^37^ Department of Neurology, Osaka University Graduate School of Medicine, Osaka, Japan

^38^ Parkinson Disease Research, Education, and Clinical Center, Philadelphia Veteran Affairs Medical Center, Philadelphia, PA, USA; Department of Neurology, University of Pennsylvania, Philadelphia, PA, USA

^39^ Department of Neurology, NYU School of Medicine, New York, NY, USA

^40^ University of Lund, Faculty of Medicine, Lund, Sweden

^41^ Movement Disorders Unit, Sant Pau Hospital and Biomedical Research Institute (IIB-Sant Pau), Barcelona, Spain

^42^ Neurology, National Hospital for Neurology & Neurosurgery, London, United Kingdom

^43^ Newcastle Magnetic Resonance Centre & Positron Emission Tomography Centre, Newcastle University, Campus for Ageing & Vitality, Newcastle upon Tyne, United Kingdom

^44^ Department of Neurology, Helsinki University Hospital, and Department of Neurological Sciences (Neurology), University of Helsinki, Helsinki, Finland

^45^ Research Institute of McGill University Health Centre, Montréal, Canada

^46^ Movement Disorders Clinic, National Institute of Neurology and Neurosurgery, Mexico City, Mexico

^47^ Department of Neurology and Psychiatry, University of Santo Tomas Hospital, Manila 1008, Philippines; International Institute of Neuroscience, Saint Luke's Medical Center, Philippines; Center for Neurodiagnostic and Therapeutic Services, Metropolitan Medical Center, Manila 1000, Philippines

^48^ Department of Clinical Neurosciences, University College London (UCL) Institute of Neurology, Royal Free Campus, Rowland Hill Street, London, UK

^49^UCL Institute of Neurology, London, United Kingdom

^50^ Department of Neurology, Northwestern University, Feinberg School of Medicine, Chicago, IL , USA

^51^ University and Institute for Research and Medical Care, IRCCS San Raffaele, Rome, Italy

^52^ Division of Neurodegenerative Diseases, Department of Neurology, Dresden University of Technology, Dresden, Germany Department of Neurology, Dresden University of Technology, Dresden, Germany German Center for Neurodegenerative Diseases (DZNE), Research Site Dresden, Dresden, Germany

^53^ UCLA/West LA VA, Los Angeles, CA, United States

^54^ Cedars-Sinai Medical Center, Los Angeles, CA, United States

^55^ Department of Neuroscience, Biomedicine, and Movement, University of Verona, Verona, Italy

^56^ Department of Pathology & Laboratory Medicine, University of Pennsylvania, Philadelphia, PA, USA; Department of Neurology, Houston Methodist Hospital, Houston, TX, USA

^57^ Department of Neurology, Fukuoka University, Japan

^58^ Northumbria Healthcare NHS Foundation Trust, North Tyneside General Hospital, Rake Lane, North Shields, Tyne and Wear, United Kingdom

^59^ Department of Psychiatry and Department of Neurology, University of Pennsylvania School of Medicine, Philadelphia, PA, USA; Parkinson's Disease and Mental Illness Research, Education and Clinical Centers, Philadelphia Veterans Affairs Medical Center, Philadelphia, PA, USA
