# Supplementary material for: Gender gap in deep brain stimulation for Parkinson’s disease
Source: NPJ Parkinsons Dis. 2022 Apr 20;8:47. doi: 10.1038/s41531-022-00305-y (PMC9021281; doi:10.1038/s41531-022-00305-y)
Supplement: Supplementary file 2 — Reporting Summary [file 41531_2022_305_MOESM2_ESM.pdf]

nature portfolio

Corresponding author(s): Dr. Stefanie T. Jost and Dr. Haidar S. Dafsari

Last updated by author(s): Nov 6, 2021

Reporting Summary

Nature Portfolio wishes to improve the reproducibility of the work that we publish. This form provides structure for consistency and transparency in reporting. For further information on Nature Portfolio policies, see our Editorial Policies and the Editorial Policy Checklist.

Statistics

For all statistical analyses, confirm that the following items are present in the figure legend, table legend, main text, or Methods section.

n/a	Confirmed	
☐	☒	The exact sample size (n) for each experimental group/condition, given as a discrete number and unit of measurement
☐	☒	A statement on whether measurements were taken from distinct samples or whether the same sample was measured repeatedly
☐	☒	The statistical test(s) used AND whether they are one- or two-sided<br>*Only common tests should be described solely by name; describe more complex techniques in the Methods section.*
☐	☒	A description of all covariates tested
☐	☒	A description of any assumptions or corrections, such as tests of normality and adjustment for multiple comparisons
☐	☒	A full description of the statistical parameters including central tendency (e.g. means) or other basic estimates (e.g. regression coefficient) AND variation (e.g. standard deviation) or associated estimates of uncertainty (e.g. confidence intervals)
☐	☒	For null hypothesis testing, the test statistic (e.g. F, t, r) with confidence intervals, effect sizes, degrees of freedom and P value noted<br>*Give P values as exact values whenever suitable.*
☒	☐	For Bayesian analysis, information on the choice of priors and Markov chain Monte Carlo settings
☒	☐	For hierarchical and complex designs, identification of the appropriate level for tests and full reporting of outcomes
☐	☒	Estimates of effect sizes (e.g. Cohen's d, Pearson's r), indicating how they were calculated

Our web collection on statistics for biologists contains articles on many of the points above.

Software and code

Policy information about availability of computer code

Data collection	The database was established in SPSS version 25 (IBM).
Data analysis	All statistical analyses were conducted with SPSS version 25 (IBM). Propensity score matching was computed with a toolbox published by Felix Thoemmes (Propensity Score Matching in SPSS. arXiv:12016385. 2012, version 3.04).

For manuscripts utilizing custom algorithms or software that are central to the research but not yet described in published literature, software must be made available to editors and reviewers. We strongly encourage code deposition in a community repository (e.g. GitHub). See the Nature Portfolio guidelines for submitting code & software for further information.

Data

Policy information about availability of data

All manuscripts must include a data availability statement. This statement should provide the following information, where applicable:

- Accession codes, unique identifiers, or web links for publicly available datasets
- A description of any restrictions on data availability
- For clinical datasets or third party data, please ensure that the statement adheres to our policy

The data used to support the findings of this study are available from the corresponding authors upon reasonable request.

Field-specific reporting

Please select the one below that is the best fit for your research. If you are not sure, read the appropriate sections before making your selection.

☒ Life sciences ☐ Behavioural & social sciences ☐ Ecological, evolutionary & environmental sciences

For a reference copy of the document with all sections, see nature.com/documents/nr-reporting-summary-flat.pdf

Life sciences study design

All studies must disclose on these points even when the disclosure is negative.

Sample size	We analyzed the number of women and men in the last five years (1) referred for DBS indication evaluations, (2) receiving positive/negative assessments, and (3) undergoing DBS surgery. In the longitudinal analysis
Data exclusions	Patients were not eligible for DBS treatment if they suffered from clinically relevant psychiatric diseases or neuropsychological impairments as assessed by a multidisciplinary team of specialized neurologists, neuropsychologists, stereotactic neurosurgeons, psychiatrists, speech and physiotherapists. In the longitudinal cohort, all patients received bilateral STN-DBS.
Replication	The findinds of this study were not replicated as this was a "real-life" study in a cohort of patients with Parkinson's disease undergoing deep brain stimulation.
Randomization	We used propensity scores to identify a sub-cohort which was precisely matched for these variables and, thereby, establish a quasi-experimental design to confirm results of the original cohort. While propensity score matching has advantages as a method providing a „pseudo-randomization‟ in observational studies, it cannot replace a randomized clinical trial. However, in certain scenarios, such as in our database, the real-life presentation of female and male PD patients may be of scientific interest.
Blinding	Blinding was not possible as this was a "real-life" study in a cohort of patients with Parkinson's disease undergoing deep brain stimulation.

Reporting for specific materials, systems and methods

We require information from authors about some types of materials, experimental systems and methods used in many studies. Here, indicate whether each material, system or method listed is relevant to your study. If you are not sure if a list item applies to your research, read the appropriate section before selecting a response.

Materials & experimental systems

n/a	Involved in the study	
☒	☐	Antibodies
☒	☐	Eukaryotic cell lines
☒	☐	Palaeontology and archaeology
☒	☐	Animals and other organisms
☐	☒	Human research participants
☐	☒	Clinical data
☒	☐	Dual use research of concern

Methods

n/a	Involved in the study	
☒	☐	ChIP-seq
☒	☐	Flow cytometry
☒	☐	MRI-based neuroimaging

Human research participants

Policy information about studies involving human research participants

Population characteristics	This was a study in female and male patients with Parkinson's disease undergoing deep brain stimulation.
Recruitment	We analyzed cross-sectional data from patients undergoing deep brain stimulation for Parkinson's disease at the University Hospital Cologne and longitudinal data from the prospective, observational, multicenter international NILS study including centers in Cologne, Marburg, Greater Manchester, and London.
Ethics oversight	Local ethics committees master votes were for Germany: Cologne #12/145, and for UK: NRES South-East London REC3-10/H0808/141 #10084.

Note that full information on the approval of the study protocol must also be provided in the manuscript.

Clinical data

Policy information about clinical studies

All manuscripts should comply with the ICMJE guidelines for publication of clinical research and a completed CONSORT checklist must be included with all submissions.

Clinical trial registration	German ClinicalTrials Register DRKS00006735.
Study protocol	https://www.drks.de/drks_web/navigate.do?navigationId=trial.HTML&TRIAL_ID=DRKS00006735
Data collection	A total sample size of 505 consisted of 316 consecutively referred patients for DBS indication evaluation at the University Hospital Cologne (01/2015–09/2020) and 189 consecutively treated patients at DBS centers in the University Hospitals Cologne and Marburg, Salford‟s Royal Hospital Manchester, and King‟s College Hospital London.
Outcomes	In the cross-sectional cohort, we examined gender proportions at referral, indication evaluations, and DBS surgery. In the longitudinal cohort, clinical assessments at preoperative baseline and 6-month follow-up after surgery included the PDQuestionnaire-8, NMSScale, Scales for Outcomes in PD-motor scale, and levodopa-equivalent daily dose. Propensity score matching resulted in a pseudo-randomised sub-cohort balancing baseline demographic and clinical characteristics between female patients and male controls.
